# Commentary: Cardiovascular Outcome in Patients Treated With SGLT2 Inhibitors for Heart Failure: A Meta-Analysis

**DOI:** 10.3389/fcvm.2021.778284

**Published:** 2021-10-26

**Authors:** Mei Qiu, Li-Min Zhao

**Affiliations:** Department of General Medicine, Shenzhen Longhua District Central Hospital, Shenzhen, China

**Keywords:** SGLT2 inhibitors (gliflozins), heart failure, NYHA class, region, race, HFPEF, HFrEF–heart failure with reduced ejection fraction

## Introduction

In the meta-analysis of Gager et al. (1) recently published in the journal *Frontiers in Cardiovascular Medicine*, the authors identified that sodium-glucose co-transporter-2 (SGLT2) inhibitors could reduce heart failure (HF) events and all-cause mortality in patients with HF and that the benefit of this drug class on the primary endpoint (i.e., a composite of hospitalization for HF HHF) or cardiovascular mortality (CVM) was consistent across relevant HF subgroups defined by the following clinically important factors: the status of type 2 diabetes at baseline, type of HF (according to left ventricular ejection fraction, LVEF), cause of HF, specific SGLT2 inhibitors, gender, age, estimated glomerular filtration rate (eGFR), body mass index, and concomitant medications. However, Gager et al. (1) failed to evaluate the effect of SGLT2 inhibitors in several subgroups defined by three other clinically important factors: race, region, and baseline New York Heart Association (NYHA) class. Hence, we aimed to conduct another meta-analysis to examine whether these factors affect the efficacy of gliflozins in patients with HF or not.

Moreover, Gager et al. in their meta-analysis (1), failed to include the latest EMPEROR-Preserved trial (2) assessing empagliflozin in patients with HF with an LVEF of > 40%. Since that trial (2) provided the new data in the subgroup of HF with mildly reduced LVEF (HFmrEF) and the subgroup of HF with preserved LVEF (HFpEF), we repeated the subgroup analysis according to LVEF by adding these new data, although this subgroup analysis had been performed in the meta-analysis of Gager et al. (1).

## Methods and Findings

We only included in this meta-analysis large cardiovascular outcome trials (CVOTs) that compared the HF outcomes of SGLT2 inhibitors with those of placebo in patients with HF. The only endpoint of interest for this meta-analysis was the composite HF outcome, which was defined as a composite of HHF or CVM. Embase and PubMed were searched (from inception to August 31st, 2021), using the following retrieval terms: “heart failure,” “HFpEF,” “HF with reduced LVEF (HFrEF),” “SGLT2 inhibitors,” “empagliflozin,” “canagliflozin,” “ertugliflozin,” “dapagliflozin,” “sotagliflozin,” and “randomized controlled trial.” Finally, we included four CVOTs (2–5) focusing on assessing gliflozins in patients with HF. We extracted trial-level survival data (i.e., hazard ratios, HRs, and 95% confidence intervals, CIs) in various subgroups of interest from included trials, and, based on them, performed a random-effects meta-analysis. Subgroup analyses were conducted according to the following four factors: region (Asia, Latin America, North America, and Europe), race (White, Asian, and Black), baseline NYHA class (Class II and Class III or IV), and LVEF at baseline (LVEF ≤ 40%, i.e., HFrEF, LVEF > 40 to <50%, i.e., HFmrEF, and LVEF ≥ 50%, i.e., HFpEF). Subgroup differences were examined by Cochran's Q test, with *P* < 0.05 indicating statistical significance. We completed data analyses using Stata (version 16.0).

**Figure 1:**
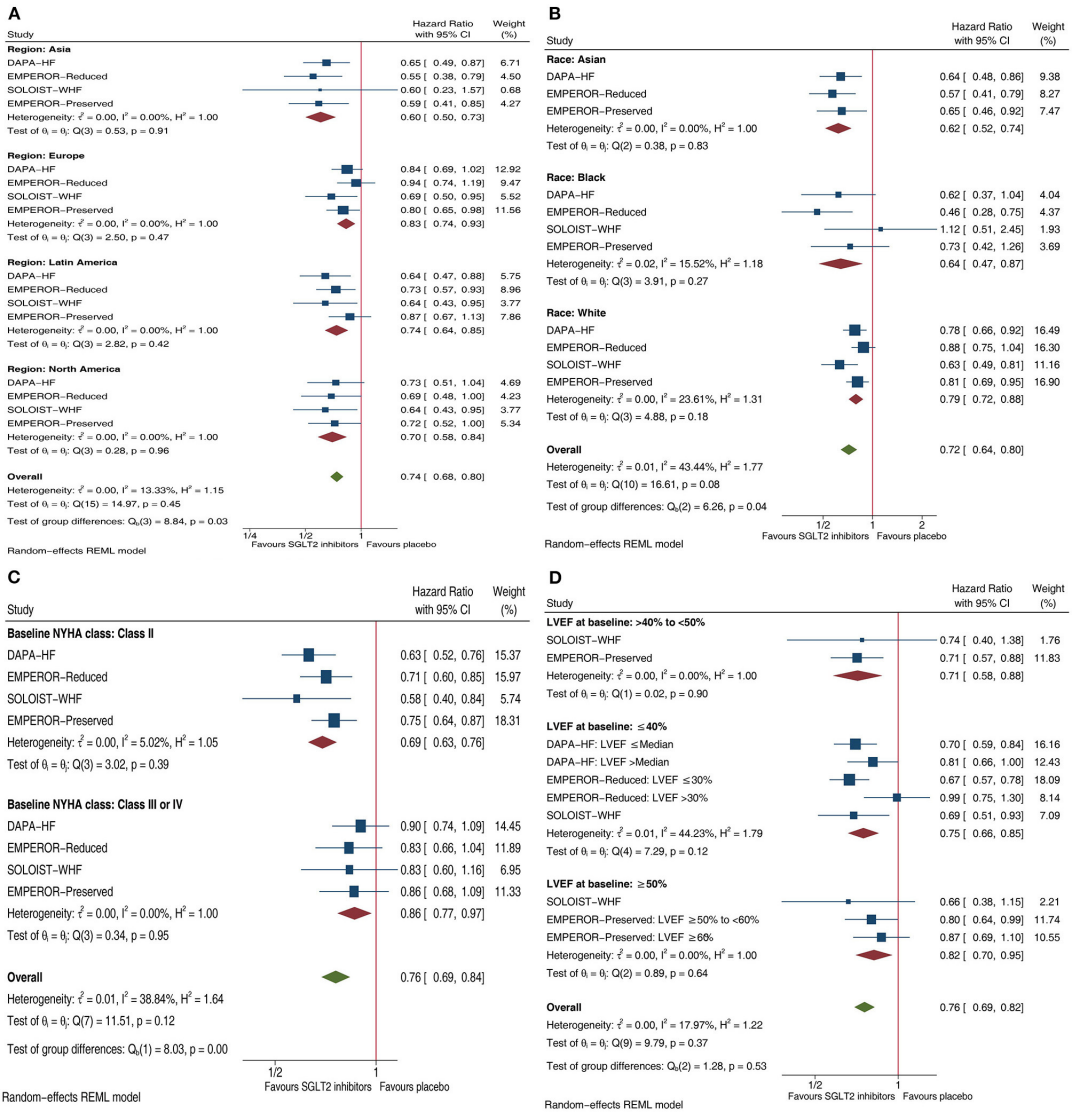
Forest plots illustrating the effect of SGLT2 inhibitors on composite HF outcome in patients with HF by four important factors. **(A)** Effects of SGLT2 inhibitors on composite HF outcome in HF patients by Regions. **(B)** Effects of SGLT2 inhibitors on composite HF outcome in HF patients by Race. **(C)** Effects of SGLT2 inhibitors on composite HF outcome in HF patients by Baseline NYHA class. **(D)** Effects of SGLT2 inhibitors on composite HF outcome in HF patients by LVEF at baseline. SGLT2, sodium-glucose co-transporter-2; HF, heart failure; CI, confidence interval; NYHA, New York Heart Association; LVEF, left ventricular ejection fraction; Composite HF outcome: defined as a composite of hospitalization for HF or cardiovascular mortality.

Compared with placebo, SGLT2 inhibitors reduced the composite HF outcome by 40% in patients with HF enrolled in Asia (HR.6, 95% CI 0.50–0.73), 17% in those enrolled in Europe (HR 0.83, 95% CI 0.74–0.93), 26% in those enrolled in Latin America (HR 0.74, 95% CI 0.64–0.85), and 30% in those enrolled in North America (HR 0.7, 95% CI 0.58–0.84); and yielded more reductions in those enrolled in Asia than in the other three continents (P_subgroup_ = 0.03; Figure 1A). SGLT2 inhibitors yielded more reductions in that outcome in Asian patients (38% reduction, HR 0.62, 95% CI 0.52–0.74) and Black patients (36% reduction, HR 0.64, 95% CI 0.47–0.87) than in White patients (21% reduction, HR 0.79, 95% CI 0.72–0.88), with a significant subgroup difference (P_subgroup_ = 0.04; Figure 1B). SGLT2 inhibitors yielded more reductions in that outcome in patients with NYHA class II (31% reduction, HR 0.69, 95% CI 0.63–0.76) than NYHA classes III–IV (14% reduction, HR 0.86, 95% CI 0.77–0.97), with a significant subgroup difference (P_subgroup_ < 0.01; Figure 1C). SGLT2 inhibitors reduced that outcome by 24% (HR 0.76, 95% CI 0.69–0.82) whether in patients with HFmrEF, patients with HFrEF patients, or patients with HFpEF patients (P_subgroup_ = 0.53; Figure 1D).

## Discussion

The meta-analysis of Gager et al. (1) confirmed the consistent efficacy of SGLT2 inhibitors on the composite HF outcome (i.e., a composite of HHF/CVM) in various HF subgroups defined by several important factors, such as baseline diabetes status, type of SGLT2 inhibitors, and baseline level of eGFR. However, Gager et al. (1) failed to perform subgroup analyses according to three other important factors: region, race, and baseline NYHA class. Thus, their findings (1) could not guide whether SGLT2 inhibitors should be used in patients with HF patients living in a specific region, those with a specific race, or those with a specific NYHA class. Furthermore, Gager et al. (1) also failed to include the latest CVOT of gliflozins, namely, EMPEROR-Preserved (2).

Conversely, we included all the published CVOTs focusing on comparing gliflozins with placebo in patients with HF, including the latest one (2). More importantly, our meta-analysis focused on subgroup analyses according to region, race, baseline NYHA class, and baseline LVEF. Accordingly, it revealed that SGLT2 inhibitors vs. placebo significantly reduced the composite HF outcome regardless of these four factors, while SGLT2 inhibitors might yield more reductions in that outcome in patients with HF living in Asia, Asian and Black patients with HF, and HF patients with NYHA class II. These findings suggest that as for preventing HF events, SGLT2 inhibitors should be recommended in patients with HF regardless of region, race, baseline NYHA class, and baseline LVEF, and especially in Asian and Black patients with HF and those with NYHA class II. Moreover, the ongoing DELIVER trial (NCT03619213) will further determine the efficacy of SGLT2 inhibitor dapagliflozin in patients with HFpEF or HFmrEF. Due to the limited number of patients with NYHA classes III–IV among included trials, further clinical trials for such a specific population may be beneficial.

In summary, based on the findings from the meta-analysis of Gager et al. (1) and ours, SGLT2 inhibitors should be recommended in a broad population of patients with HF patients, those with diabetes or not, those with reduced eGFR or not, and those with preserved/reduced LVEF. Moreover, SGLT2 inhibitors should be more recommended in Asian and Black patients with HF and patients with HF and with NYHA class II because of the greater benefits of this drug class in these HF subpopulations.

## Author Contributions

MQ: design. MQ and L-MZ: conduct/data collection, analysis, and writing manuscript. All authors contributed to the article and approved the submitted version.

## Conflict of Interest

The authors declare that the research was conducted in the absence of any commercial or financial relationships that could be construed as a potential conflict of interest.

## Publisher's Note

All claims expressed in this article are solely those of the authors and do not necessarily represent those of their affiliated organizations, or those of the publisher, the editors and the reviewers. Any product that may be evaluated in this article, or claim that may be made by its manufacturer, is not guaranteed or endorsed by the publisher.
